# Keyword-augmented and semi-automatic generation of FESS reports: a proof-of-concept study

**DOI:** 10.1007/s11548-022-02791-0

**Published:** 2022-11-17

**Authors:** V. Kunz, V. Wildfeuer, R. Bieck, M. Sorge, V. Zebralla, A. Dietz, T. Neumuth, M. Pirlich

**Affiliations:** 1 Department of Otolaryngology, Head and Neck Surgery, University Hospital Leipzig, Liebigstraße 10-14, 04103 Leipzig, Germany; 2 Innovation Center Computer Assisted Surgery (ICCAS), University of Leipzig, Leipzig, Germany

**Keywords:** Artificial intelligence, Deep learning, Natural language processing, Neural language models, FESS, Surgical reports

## Abstract

**Introduction:**

Surgical reports are usually written after a procedure and must often be reproduced from memory. Thus, this is an error-prone, and time-consuming task which increases the workload of physicians. In this proof-of-concept study, we developed and evaluated a software tool using Artificial Intelligence (AI) for semi-automatic intraoperative generation of surgical reports for functional endoscopic sinus surgery (FESS).

**Materials and methods:**

A vocabulary of keywords for developing a neural language model was created. With an encoder-decoder-architecture, artificially coherent sentence structures, as they would be expected in general operation reports, were generated. A first set of 48 conventional operation reports were used for model training. After training, the reports were generated again and compared to those before training. Established metrics were used to measure optimization of the model objectively. A cohort of 16 physicians corrected and evaluated three randomly selected, generated reports in four categories: “quality of the generated operation reports,” “time-saving,” “clinical benefits” and “comparison with the conventional reports.” The corrections of the generated reports were counted and categorized.

**Results:**

Objective parameters showed improvement in performance after training the language model (*p* < 0.001). 27.78% estimated a timesaving of 1–15 and 61.11% of 16–30 min per day. 66.66% claimed to see a clinical benefit and 61.11% a relevant workload reduction. Similarity in content between generated and conventional reports was seen by 33.33%, similarity in form by 27.78%. 66.67% would use this tool in the future. An average of 23.25 ± 12.5 corrections was needed for a subjectively appropriate surgery report.

**Conclusion:**

The results indicate existing limitations of applying deep learning to text generation of operation reports and show a high acceptance by the physicians. By taking over this time-consuming task, the tool could reduce workload, optimize clinical workflows and improve the quality of patient care. Further training of the language model is needed.

**Supplementary Information:**

The online version contains supplem- entary material available at 10.1007/s11548-022-02791-0.

## Introduction

Artificial Intelligence (AI) represents the continuous digital development of our environment, especially in the health sector, where the number of applications is expected to increase in the future. In 2020, the AI market within the healthcare system had an estimated value of around 5.9 billion US-Dollar [1]. Still controversial remains the question whether AI developments will fundamentally revolutionize the healthcare system or even replace entire areas of it. It is known that these applications can solve complex and time-consuming problems in the clinical routine. So far, AI is not broadly established and consistently implemented in the healthcare sector [2]. However, a large spectrum of AI-based applications exists in development and clinical trials that are closely related to medical imaging, e.g., in diagnostic radiology [3, 4]. Conversely, there is still far-reaching potential for the specific field of surgery and, respectively, surgical procedures—as explained in the following.

One sub-genre of AI is machine learning, which currently receives the most attention in research. Specifically, computer vision (CV) and Natural Language Processing (NLP) are core algorithm domains that solve complex tasks such as object detection and text analysis. NLP allows technologies to understand and interpret natural language for transferring unstructured data into structured and machine-understandable information [2, 5, 12]. The implementation of CV and NLP applications could change and improve the clinical routine of many fields within the healthcare system, especially in otorhinolaryngology (ORL) [2]. It was shown that AI applications, in general, could decrease the workload and optimize the workflow of physicians by overtaking complex tasks, e.g., automatically generated radiology reports by analyzing medical images with connected natural language descriptions [5].

Usually, surgical reports are written in free text form after the actual procedure by the performing surgeons is finished. Some authors reported advantages of standard text templates for the creation of reports compared to conventionally using free-form reports [6]. Due to the difficulty of writing reports from memory—at some points hours after the actual procedure—this task is error-prone and time-consuming. Considering this, an application producing detailed, individual reports during the procedure might be a relevant contribution to optimizing the clinical workflow. NLP-based approaches where text keywords relate to a specific part of the procedure to produce coherent sentence structures already exist. These enable the generation of a complete and semantically logical surgical report. Regarding that topic, improvements in the field of image- and text-based production of reports based on NLP and deep learning processes were shown [4, 7]. Those previous works set the foundation for generating radiology reports (4) and prediction of navigation steps during endoscopic operations by automatically analyzing image structures and datasets (7). The ability of “intelligent” technologies to generate reports by analyzing images or interpreting natural language like keywords is used in this work.

In this proof-of-concept study, an NLP tool was implemented to generate semi-automatically surgical reports based on existing reports of functional endoscopic sinus surgery (FESS) and keywords recorded during surgery. This study highlights the potential that a neural language model offers to produce complete and coherent surgical reports along with the actual procedure. Using this development, the daily workload of physicians could be reduced, and clinical workflows might be optimized. Furthermore, this AI application might increase physicians’ time for patient care and treatment.

## Materials and methods

### Neural language model and generation of surgical reports

In this proof-of-concept study, a machine-interpretable vocabulary was created for documented FESS procedures and enclosed in a neural language model. Specific keywords were defined and assigned to steps of the FESS procedure so that the model could create coherent sentence structures as they would be expected in conventional surgical reports. Therefore, 48 anonymized conventional reports of FESS, written by ten experienced surgeons, were retrospectively included in the model's training. These reports had a length of 10 to 45 sentences and provided the baseline training data. Thus, a tool with the ability to artificially generate a coherent report from conventional text modules was created.

Subsequently, a database with 1500 sentences out of the 48 reports and an average number of 8 words per sentence was set up. In the first step, a vocabulary of 150 keywords relating to a specific part of the surgical reports was defined with the help of medical experts to create a sentence of anchor words, which are likely to be used as comments by the operating surgeons during the procedure. These words were enclosed in the model for training. Furthermore, the keywords or keyword combinations were matched with the corresponding sentences within the surgical reports through an annotation step by two experts. In this way, 2 to 4 keywords were assigned to each sentence. Respectively, two consecutive sentences were paired to create individual training patterns. These patterns consist of an initial sentence, a target sentence, and the specific keywords of the target sentence, which were assigned to the associated initial sentence.

The prepared sentence pairs were tokenized for obtaining a numeric representation for model training. We decided to train an unsupervised word-tokenizer with *SentencePiece* [8], as it provides several benefits for generating surgical reports. Our tokenizer uses a fixed vocabulary size of *s* = 500, which is set before training the language model. This assures that the trained model is independent of language-specific aspects like the openness of the vocabulary. Additionally, an augmentation step is integrated to virtually increase database variability and improve the robustness of the model in the context of imbalanced word occurrence.

For the language model, we used an *encoder-decoder-architecture* which consists of bidirectional 2-layer-LSTMs (long short-term memory) for both coders. The encoder transforms the tokenized input sentences into a continuous intermediate representation, which is also known as *latent features*. The decoder further generates an output sentence word by word based on the previously generated word and these latent features of the input sentence. The decoder model is autoregressive and uses the last generated output word as an input word for generating the following word in the sentence so that the conditional probability can be maximized. The primary assumption of the language model is that surgical report sentences follow an intrinsic word distribution typical for reports of the ORL-Department. The language model training maximizes the probability of generating the correct word combination for the new reports by emulating this specific word distribution.

Additionally, we used the attention pattern by Chan et al. [9], which calculates a weighted context between the latent features of the encoder and the already generated output words for each step of the word generation. This enables the possibility that the decoder can respond to individual, word-specific dependencies between the input and the output sentence more accurately. Both the encoder and the decoder have a word embedding size of 512, which translates our numeric word representation into a vector representation optimized for GPU-based training.

For the model training, k-fold cross-validation with *k* = 10 was used for our 48 datasets of surgical reports. The report sentences of the left-in-datasets were combined into one dataset, shuffled, and divided into training and validation sentences with a ratio of 85:15. In the first step of the training pipeline, we artificially enlarged the data with a sequence of random sentence augmentations by swapping, deletion, addition, synonym and insertion operations in a sentence. Our sentence encoder was pretrained on the German CC100 dataset to offset the limitations of the currently small corpus of procedure-specific keywords.

**Fig. 1:**
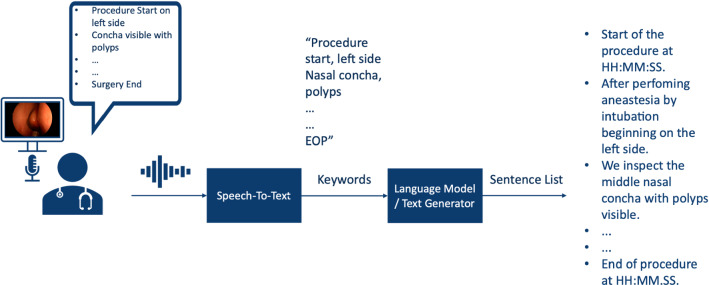
Operating scheme of the NLP-assisted tool for generating surgical reports

To outline the effectiveness of the training process, model results were generated and compared before and after the training with objective machine translation metrics used in NLP literature, ROUGE, COSS, and METEOR [10]. All metrics were calculated by comparing generated and original report sentences with each other. BLEU (bilingual evaluation understudy) describes the sentences’ specificity within a range of 0–1. We employed BLEU-2 as the metric variation that allows us to compare how many two-word pairs in the generated reports match with the original sentences. Additionally, ROUGE (recall-oriented understudy for gisting evaluation) has been chosen to describe the sentences’ sensitivity within a range of 0–1. This metric also identifies how many words in the generated sentences are also found in the original sentence. The cosine similarity metric (COSS) is a custom metric that describes the similarity based on angular distance between two vectors in space. We used the vectors generated in our word embedding layers of the model representing a word in a vector with 500 values). The COSS is the mean angular distance between each word of the generated sentence and the original one with a range of 0–1. A value of 0 indicates an orthogonal relationship with the lowest semantic similarity, while a value of 1 would indicate maximum word similarity. Lastly, we chose the METEOR metric (metric for evaluation of translation with explicit ordering) that describes the F1-value of the sentences with more focus on specificity and semantics. The metric generates values in a range between 0 and 1. The conventional surgical reports were validated as the standard with a value of 1.0. Figure 1 displays a summary of the AI-assisted tool for generating surgical reports.

### Study participants and data acquisition

For the evaluation of the artificially generated reports, 18 Otorhinolaryngologists, trainees and consultants in equal parts, were recruited. Thereby, only those who at least had clinical-theoretic knowledge of FESS were included, ensuring they were able to understand the possible benefits and optimization of clinical workflows by using this tool. Each physician received three AI-augmented, artificially generated reports with the related conventional one, as well as an accompanying task explanation and a link for the online evaluation via e-mail. For this first evaluation, three representative conventional reports were disassembled into specific keywords. On this basis, the tool generated new reports like it would perform during an operation with speaking in these keywords.

The surgical reports, including the editing information of the participants, were sent back to us via e-mail. Before analyzing the data, the documents were anonymized. This study was conducted under the approval granted by the University ethics committee (ethics approval: *455/20-ek*).

### Evaluation of the artificially generated reports

First, the participants were asked to correct three artificially generated reports until they were considered appropriate surgical reports. For the subsequent analysis of the corrections, all edits in the documents were counted and categorized using *Microsoft Word*®. Then, the evaluation was accomplished online using a questionnaire with 13 questions created for this study, which was divided into the following four categories: (1) “quality of the generated surgical reports”, (2) “time-saving”, (3) “clinical benefits” and (4) “comparison with the conventional surgical reports”. In the first category, grammar, content, and reading flow of the artificially generated surgical reports had to be evaluated using school grades according to the German grading system. In the second category, the participants had to estimate their daily time consumption by writing conventional surgical reports after the procedure and the possible time saved by using this tool. To avoid possible distortions of the results, we reevaluated the question regarding daily time saving, as the option to select “no time saving” was not explicitly provided in a first version of the questionnaire. We replaced the original question by the reevaluated one und updated the online questionnaire. Within the third category, participants had to evaluate the “clinical benefits”, workload reduction, and their willingness to use this tool in the future. In the fourth and last category, the artificially generated and corrected reports were compared with the related conventional surgical reports concerning their form and content.

### Statistical analysis

Statistical analysis was performed using *SPSS* (*Statistical Package for the Social Sciences*, *IBM*, Version 23.0.0). Categorical data are reported as absolute and relative frequencies (proportions and percentages). Continuous data are expressed as mean (± SD). Wilcoxon’s test was used to analyze differences in objective parameters before and after the training of the language model.

## Results

In total, 18 physicians participated in this study, out of which two had to be excluded because the corrections and recorded editing of the generated surgical reports from these participants were unavailable. Out of the 16 remaining participants, 50% were ORL-consultants, and 50% were physicians in training.

### Corrections of artificially generated reports

The type and number of corrections done by the participants were recorded and evaluated (Table 1). An average of 23.25 corrections were necessary until the reports were subjectively considered appropriate surgical reports by the participants.

**Table 1:** Number of corrections after editing the artificially generated surgical reports

	Elements added	Elements removed	Grammar mistakes	Sentences added	Sentences removed	Corrections total
Corrections	9.3 ± 6.5	13.9 ± 7.2	13.4 ± 5.9	5.4 ± 4.8	8.1 ± 5.3	23.3 ± 12.5
Minimum	1	6	4	0	3	8
Maximum	26	32	23	14	18	58

### Grading of grammar, content, and reading flow

The participants gave an average grade of 3.1 for the category “grammar”, an average grade of 2.8 for “content,” and an average grade of 2.6 for “reading flow”.

### Daily expenditure and possible saving of time

66.67% of physicians estimated a time-expenditure of about 30–60 min per day for conventional surgical reports after the actual procedure was performed. In a more detailed reevaluation, a majority of 61.11% estimated that 16–30 min per day can be saved by using this AI-assisted tool for generating surgical reports (Table 2). 27.78% guessed a timesaving of 1–15 min and 5.56% of 31–45 min per day (Table 2).

**Table 2:** Estimated time generating conventional reports and time saved* using the AI-assisted tool

Expenditure of time for surgical reports (minutes/day)		0–30		30–60	
Participant’s choice (%)		33.33		66.67	
Time saved using the AI-assisted tool (minutes/day)	0	1–15	16–30	31–45	46–60
Participant’s choice* (%)	5.56	27.78	61.11	5.56	0.00

*Reevaluated question

### Keyword-augmented training of the language model

**Fig. 2:**
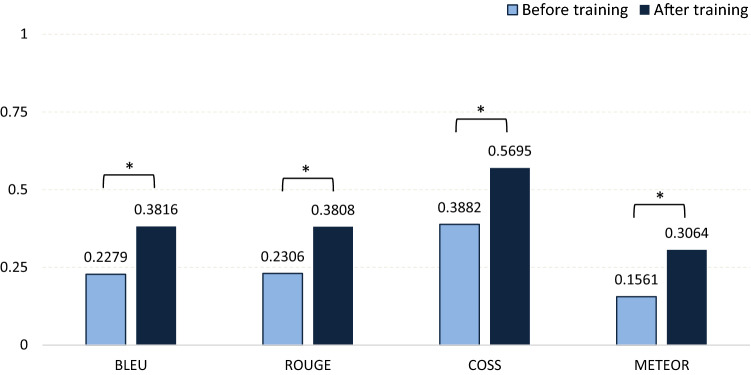
Wilcoxon’s test for objective parameters of the neural language model

Objective parameters from 48 FESS reports were compared before and after training. The first metric BLEU increased with a Z_BLEU_ of − 4.09, and ROUGE increased with a Z_ROUGE_ of − 3.84, COSS with a Z_COSS_ of − 4.61, and METEOR with a Z_METEOR_ of − 3.90. The results of Wilcoxon’s test show that the keyword-augmented training and optimization of the language model significantly (*p* < 0.001) increased all objective parameters (Fig. 2).

### Clinical evaluation

**Fig. 3:**
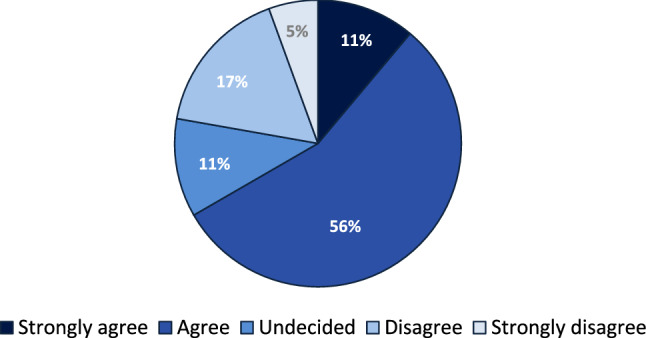
Pie chart of willingness to use the AI-assisted tool in the future

The results show that 61.11% of the study participants expected a workload reduction by using this tool for generating surgical reports. Clinical benefits were seen by 66.66% of the participating physicians. 33.33% claimed that there is a similarity in the content between the generated and corrected reports compared to the conventionally generated ones. 27.78% of the participants agreed that there is a formal similarity between the generated and corrected reports compared to the conventionally generated ones (Table 3 and Fig. 3).

**Table 3:** Evaluation of advantages and content-related parameters of this tool

Parameters	Strongly agree (%)	Agree (%)	Undecided (%)	Disagree (%)	Strongly disagree (%)
Workload reduction	27.78	33.33	16.67	16.67	5.56
Clinical benefits	22.22	44.44	16.67	11.11	5.56
Would use tool in future	11.11	55.56	11.11	16.67	5.56
Strongly similar in content	0.00	33.33	50	11.11	5.56
Strongly similar in form	0.00	27.78	33.33	38.89	0.00

## Discussion

Implementing AI-based applications in clinical work is becoming more important [2–4]. Those technologies partially adopt repetitive and time-consuming tasks to reduce the physicians' workload and standardize and optimize the quality of surgical reports. Within this study, we developed and evaluated a neural language model for the semi-automatic generation of surgical reports using the example of FESS.

### Number of corrections

The extent of corrections among the study participants varied considerably. This might either be caused by different levels of training between residents and consultants, or due to individual adjustments and preferences of the participants. More studies are needed to further investigate the reason behind these findings. The total number of corrections indicates that there is still a need of editing the generated reports after the surgical procedure. Further training with more reports is necessary to optimize the generated reports' quality to reduce the time of correcting the reports after the procedure [2].

### Daily expenditure and possible saving of time

The results of the reevaluation show that 27.78% of the participants are expecting a daily time saving of 1–15 min and 61.11% of 16–30 min, which indicates an important aspect for reducing the workload and optimizing the clinical workflow [5]. Through taking over repetitive and time-consuming tasks with these applications, such as writing reports [11], physicians can potentially shift valuable time to patient treatment [5, 12–14]. Thus, the quality and safety of patient care might improve as well, while mortality may decrease in this context [5]. Furthermore, these results indicate, that there is an acceptance of our tool by the participants, which increases the chance for implementation in clinical routine.

### Metrics

For the objective evaluation of our model, we used metrics to assess neural machine translation. These parameters were used to validate the reports before and after the training. The results prove that the generated reports can improve significantly (*p* < 0.001) through the training model. This language model generates the reports based on the keywords and is also oriented to word order. Consequently, the most likely sentence is generated as it would be expected in a surgical report. By enlarging the training data set of more conventional reports, the model may generate more accurate and high-quality surgical reports before integration into a daily clinical routine [2].

### Evaluation

About 66.66% of the participants see benefits, while 61.11% see a relevant workload reduction through this tool. Furthermore, 66.67% of the attended surgeons would use this tool for generating surgical reports in the future. On the one hand, these results show a great acceptance for implementing those new technological applications in the clinical workflow [6]. On the other hand, this emphasizes the requirement to revolutionize the repetitive and time-consuming task of writing surgical reports. This is in line with the review by Bur et al., who describe the future major impact of AI and the need to understand its principles and scopes, especially in ORL practice and documentation [2]. The similarity of form and content between the generated and the related conventional reports (considered as “gold-standard”), seen by about 30% of the participants, indicates a satisfying performance of the tool, which is continuously improving. Thus, the generated reports will soon be brought progressively in line with the conventional reports concerning form and content.

### Limitations

There are some obstacles to overcome before this AI tool can be used in clinical practice in the future. First, the surgeons must be taught how to handle this keyword-based application to avoid misconceptions. Second, the surgeons using this tool must, at the moment, wear a microphone during the operation procedure to speak in their keywords. This has already been realized for education purposes. Furthermore, the language model must be able to distinguish between the keywords and outer-operative dialogs, as this could distort the content of the generated reports significantly. The conventional reports written by experienced surgeons were used as a gold standard in clinical routine and may vary in detail, which is left up to each individual surgeon. With the help of AI-based applications, it could be possible to create a certain standard of detail and information for operation reports. Moreover, we only recruited a small first cohort of 16 physicians with clinical occupations in ORL to evaluate this proof-of-concept study. In further evaluations, larger cohorts must prove the interface and handling of this tool for facilitating a user-friendly application. In this study, trainees and consultants were included in equal parts. The consultants are more experienced in documentation, so they might take less time, but are also performing more operations per day than trainees do. Thus, the time needed for writing surgical reports should be balanced between the trainees and the consultants. In a first version of the online questionnaire, within the question regarding possible time saved per day by using this tool, the option of “no time saving” was not explicitly provided. To avoid distortions of the results, we contacted the participants again and reevaluated this question and provided more detailed options to choose for the participants. We replaced the original question by the reevaluated question und updated the online questionnaire. It is relevant to reevaluate this item in further studies under clinical conditions after optimizing the tool with enlarged datasets. The number of corrections needed to gather an acceptable operation report also shows that optimizing the quality is necessary to reduce the postoperative editing expenditure time. Therefore, well-structured conventional reports are indispensable for enlarging the training data set and optimizing the quality of the artificially generated reports [5].

### Prospects

Due to the ability of AI-based technologies to enhance themselves by learning from their successes and defeats as a feedback loop [2], it is relevant to generate a huge number of reports to improve the model's performance continuously. Further optimization and training of this AI-based application are indispensable and a continuous process. Through this, the quality and accuracy of the generated reports can and will be continuously improved. It is possible to upgrade the tool with other AI-based technologies to achieve a higher quality of reports. Therefore, we are simultaneously developing an image-based tool that can recognize and analyze endonasal endoscopic videos with the help of a pattern recognition software. That software was already reported in ORL procedures [2, 7]. Google® already uses similar technologies to provide video content information [2] automatically. Based on the images within the endoscopic video, this tool can generate a detailed operation report during the actual procedure.

Moreover, it is possible to include screenshots in the created report for accurate documentation of, for example, suspect findings. These highly-detailed reports can also have advantages for legal purposes. By combining both tools, the model can receive more information from the endoscopic video and the spoken keywords to generate more accurate and higher-quality surgical reports in the future. A further consideration might be to create several versions of one report with different levels of detail for different purposes. For example, highly detailed reports could be saved for legal purposes, and moderately detailed reports for briefly reconstructing a procedure. Another important aim of those applications could be the standardization of surgical reports for better interdisciplinary exchange and quality management [1, 5, 6, 12].

First studies describing the regular use of artificial intelligence in surgery [12], as well as automatic report generation in radiology [5] already exist. This proof-of-concept study showed that there is a need and acceptance of new AI-based applications for generating (ORL-) surgical reports. Therefore, it is important that physicians actively support this technological development [6, 12]. Finally, our AI tool can be transferred to other clinical departments.

## Conclusion

The digital revolution in healthcare is inevitable [1–3]. AI-based technologies will become an indispensable part of the clinical workflow for reducing physicians' workload and optimizing the quality and patient safety [1, 2, 5, 6, 12, 14]. The developed tool is an example and, therefore, part of the healthcare sector's digital revolution. An estimated time saving of 16–30 min per day and a relevant workload reduction was seen by most of the participants. These results point out the need of optimizing the repetitive and time-consuming task of generating surgical reports. Further training of the model is needed to improve its performance, emphasizing the necessary process of advancement in AI-based technologies for clinical application. With this proof-of-concept study, a first step in the indispensable development of medical AI technologies was made to optimize clinical workflows, possibly contributing to enhance the quality and safety of patient care in future.

## Supplementary Information

Below is the link to the electronic supplementary material.

Supplementary file1 (DOCX 15 kb)

Supplementary file2 (DOCX 24 kb)

## Data Availability

The datasets used and/or analyzed during the current study are available from the corresponding author on reasonable request.

## Abbreviations

AI: Artificial intelligence
BLEU: Bilingual evaluation understudy
COSS: Cosine similarity
FESS: Functional endoscopic sinus surgery
GPU: Graphics processing unit
IBM: International business machines corporation
LSTM: Long short-term memory
METEOR: Metric for evaluation of translation with explicit ordering
ML: Machine learning
NLP: Natural language processing
ORL: Otorhinolaryngology
p: P-value
ROUGE: Recall-oriented understudy for gisting evaluation
SD: Standard deviation
SPSS: Statistical package for the social sciences

## References

[1] Artificial Intelligence In Healthcare Market Size Report (2021), 2019–2025. https://www.grandviewresearch.com/industry-analysis/artificial-intelligence-ai-healthcare-market. Accessed 28 Feb 2021

[2] Bur AM, Shew M, New J (2019). Artificial intelligence for the otolaryngologist. A state of the art review. Otolaryngol-Head Neck Surg.

[3] Liu X, Faes L, Kale AU (2019). A comparison of deep learning performance against healthcare professionals in detecting diseases from medical imaging. A systematic review and meta-analysis. Lancet Digit Health.

[4] Monshi MMA, Poon J, Chung V (2020). Deep learning in generating radiology reports. A survey. Artif Intell Med.

[5] Letourneau-Guillon L, Camirand D, Guilbert F (2020). Artificial intelligence applications for workflow, process optimization and predictive analytics. Neuroimaging Clin N Am.

[6] Eryigit Ö, van de Graaf FW, Lange JF (2019). A systematic review on the synoptic operative report versus the narrative operative report in surgery. World J Surg.

[7] Bieck R, Heuermann K, Pirlich M (2020). Language-based translation and prediction of surgical navigation steps for endoscopic wayfinding assistance in minimally invasive surgery. Int J CARS.

[8] Kudo T (2018) Subword regularization. Improving neural network translation models with multiple subword candidates. In: Gurevych I, Miyao Y (eds) Proceedings of the 56th annual meeting of the association for computational linguistics, vol 1: long papers. Association for computational linguistics, Stroudsburg, PA, USA, pp 66–75

[9] Chan W, Jaitly N, Le QV et al. (2015) Listen, attend and spell

[10] Lin C-Y, Och FJ (2004) Automatic evaluation of machine translation quality using longest common subsequence and skip-bigram statistics. In: Scott D (ed) Proceedings of the 42nd annual meeting on association for computational linguistics—ACL '04. Association for computational linguistics, Morristown, NJ, USA, 605-es

[11] Jing B, Xie P, Xing E (2018) On the automatic generation of medical imaging reports. In: Gurevych I, Miyao Y (eds) Proceedings of the 56th annual meeting of the association for computational linguistics, vol 1: long papers. Association for computational linguistics, Stroudsburg, PA, USA, pp 2577–2586

[12] Hashimoto DA, Rosman G, Rus D (2018). Artificial intelligence in surgery promises and perils. Ann Surg.

[13] Lin SY, Shanafelt TD, Asch SM (2018). Reimagining clinical documentation with artificial intelligence. Mayo Clin Proc.

[14] Quiroz JC, Laranjo L, Kocaballi AB (2019). Challenges of developing a digital scribe to reduce clinical documentation burden. NPJ Digit Med.

