# Supplementary material for: Keyword-augmented and semi-automatic generation of FESS reports: a proof-of-concept study
Source: Int J Comput Assist Radiol Surg. 2022 Nov 17;18(5):961–8. doi: 10.1007/s11548-022-02791-0 (PMC10113317; doi:10.1007/s11548-022-02791-0)
Supplement: Supplementary file 1 — Supplementary file1 (DOCX 15 kb) [file 11548_2022_2791_MOESM1_ESM.docx]

| OP-Bericht Beispiel  Künstlich erzeugter OP-Bericht   \| OP in Intubatiosnarkose, Team-time-out, korrekter Patient/in, Aufklärung vorliegend. \| \| --- \| \| Hohe Einlage (Naphazolin/Tetracain) bds., folgend steriles Abwaschen und Abdecken. \| \| Steriles Abwaschen und Abdecken. \| \| Beginn links. Mit dem Blakesly Abtragung von etwas polypösem Gewebe aus dem Siebeinschacht und Entfernung eines Restes des Proc. uncinatus. \| \| Die Uncinektomie in gleicher Weise. \| \| Resektion reichlich polypösem Gewebe im mittleren Nasengang wird schrittweise Durchführung einer hinteren Ethmoidektomie mit entsprechender Darstellung der Schädelbasis sowie der Lamina papyracea. \| \| Darstellen der Keilbeinhöhlenvorderwand, die linksseitig sehr klein ist - hier keine weiteren Maßnahmen. \| \| Darstellung der Kieferhöhlenöffnung und Erweiterung des Kieferhöhlenostiums mittels Kieferhöhlensauger, rückwartsgreifender Stanze und Antrumstanze unter Resektion von reichlich Polypen aus der Kieferhöhle mit Kieferhöhlensauger und Heuwieser. \| \| Aufsuchen des Rec frontalis, der funktionell frei dargestellt wird. \| \| Es entleert sich massic Pus_Abstrich ad MIBI. \| \| Nun Resektionskontrolle, Ausschluss von Verletzung und polypösem Restgewebe. Keilbeinhöhle, Schädelbasis, Lamina papyracea.. \| \| Anschließend Zuwenden zur rechten Seite und Vorgehen in gleicher Weise.. \| \| Hier stellt sich die Keilbeinhöhle soweit unangetastet dar. \| \| Nach Eröffnung Entfernung von Polypengewebe. \| \| Eröffnung der Bulla ethmoidalis und des Processus uncinatus und reichlich polypösem Gewebe im mittleren Nasengang. \| \| Die Kieferhöhle weist lediglich eine Schleimhauthypertrophie auf, ausreichende und freie Öffnung. \| \| Hier keine weiteren Maßnahmen. \| \| Abschließend NaCl-Spülung beidseits und Einbringen von Predni-Ophthalgel beidseits. \| \| Jeweils eine Fingerlingstamponade beidseits unter Sicht in den entstandenen Siebbeinschacht. \| \| End_of_Document. \|  Originalbericht Team timeout. Richtiger Patient. Hohe Einlage bds., steriles Abwaschen und Abdecken. Beginn links: Mit dem Blakesly Abtragung von etwas polypösem Gewebe aus dem Siebeinschacht und Entfernung des Proc. uncinatus. Weitere Extirpation von Polypengewebe bis zum Erreichen der Schädelbasis unter videoendoskopischer Sicht. Präparation des hinteren Siebbeins unter kontinuierlicher Exstirpation von wandständigem polypösem Gewebe. Darstellen der Keilbeinhöhlenvorderwand, die linksseitig sehr klein ist - hier keine weiteren Maßnahmen. Darstellung der Kieferhöhlenöffnung und Erweiterung des Kieferhöhlenostiums mittels Kieferhöhlensauger, rückwartsgreifender Stanze und Antrumstanze unter Resektion von reichlich Polypen aus der Kieferhöhle mit Kieferhöhlensauger und Heuwieser. Aufsuchen des Rec. frontalis. Dieser wird nicht forciert, da CT-morphologisch beide Stirnhöhlen aplastisch sind. Nun Resektionskontrolle, Ausschluss von Verletzung und polypösem Restgewebe: Keilbeinhöhle, Schädelbasis, Lamina papyracea.. Zuwenden zur rechten Seite und Vorgehen in gleicher Weise. Hier stellt sich die Keilbeinhöhle soweit unangetastet dar. Nach Eröffnung Entfernung von Polypengewebe. Außerdem Ausräumung von Polypengewebe aus dem Siebbein und Stirnhöhleneingang sowie dem dorsalen mittleren Nasengang. Die Kieferhöhle weist lediglich eine Schleimhauthypertrophie auf, ausreichende und freie Öffnung. Hier keine weiteren Maßnahmen. Abschließend NaCl-Spülung beidseits und Einbringen von Predni-Ophthalgel beidseits. Je eine Fingerlingstamponade beidseits unter Sicht in den entstandenen Siebbeinschacht. End_of_Document. |
| --- | --- | --- | --- | --- | --- | --- | --- | --- | --- | --- | --- | --- | --- | --- | --- | --- | --- | --- | --- | --- |
