# Supplementary material for: Keyword-augmented and semi-automatic generation of FESS reports: a proof-of-concept study
Source: Int J Comput Assist Radiol Surg. 2022 Nov 17;18(5):961–8. doi: 10.1007/s11548-022-02791-0 (PMC10113317; doi:10.1007/s11548-022-02791-0)

Fragebogen (inkl. Reevaluation)

**Schlagwortbasierte OP-Berichterstellung mit Hilfe künstlicher Intelligenz**

Vergleichende Studie zur Evaluation von schlagwortbasierten, durch ein Computerprogramm erstellten Berichten von Nasennebenhöhlen-Operationen

* Erforderlich

1. Liebe Teilnehmer*innen, bei der Umfrage zum Thema "Schlagwortbasierte OP- Berichterstellung mit Hilfe künstlicher Intelligenz" handelt es sich um eine Befragung, in der Valentina Wildfeuer verantwortlich für den Inhalt sowie die Auswertung der Umfrage ist. Fragen zur Umfrage richten Sie gerne an: valentina.wildfeuer@medizin.uni-leipzig.de. Der Datensatz wird anonym ausgewertet und im Rahmen der gesetzlichen Aufbewahrungsfrist digital und für unbefugte unzugänglich gespeichert. Ich versichere, dass die von Ihnen erhobenen und nachfolgend benannten personenbezogenen Daten zu jeder Zeit ausreichend vor unberechtigtem Zugriff gesichert sind. Für die Teilnahme an dieser Umfrage brauchen sie sich weder registrieren, noch an anderer Stelle Ihren Namen angeben. Nehmen Sie sich nach dem Ausfüllen des Fragebogens noch einmal die Zeit, Ihre Angaben zu überprüfen und ggf. zu ändern, bevor Sie den Fragebogen absenden. Bitte beachten Sie, dass Ihnen jederzeit ein Auskunfts- und Berichtigungsrecht in Bezug auf die zu ihrer Person gespeicherten Daten zusteht. Außerdem gibt Ihnen das Datenschutzrecht die Möglichkeit, Ihre Einwilligung zur Teilnahme an dieser Befragung jederzeit zu widerrufen und die Löschung Ihrer personenbezogenen Daten zu verlangen. Nach erfolgtem Widerruf werden Ihre Daten umgehend gelöscht. Sofern Sie einen Widerruf in Erwägung ziehen, merken Sie sich bitte Datum und Uhrzeit der Fragebogenausfüllung, damit wir sicherstellen können, den richtigen Fragebogen aus dem Datensatz zu entfernen. Dasselbe gilt für die Daten (Bearbeitungszeit und Korrekturen) der Word-Dateien mit den von Ihnen bearbeiteten OP- Berichten, welche Sie per Mail an valentina.wildfeuer@medizin.uni-leipzig.de zurückschicken. *

*Markieren Sie nur ein Oval.*

- Ja, ich bin einverstanden.

**Schlagwortbasierte OP- Berichterstellung mit Hilfe künstlicher Intelligenz**

Vergleichende Studie zur Evaluation von schlagwortbasierten, durch ein Computerprogramm erstellten Berichten von Nasennebenhöhlen- Operationen

**Daten des Evaluierenden**

2. In welchem Fachbereich arbeiten Sie? *

*Markieren Sie nur ein Oval.*

- Hals-Nasen-Ohrenheilkunde
- Sonstiges: _________________

3. Was trifft auf Sie zu? *

*Markieren Sie nur ein Oval.*

- Assistenzärztin/-arzt
- Oberärztin/-arzt
- Chefärztin/-arzt
- Sonstiges:__________

**Qualität der Berichte**

Bitte beziehen Sie sich auf den Gesamteindruck der OP-Berichte nach Ihrer Korrektur.

4. Grammatik (Rechtschreibung, Zeichensetzung, Satzbau) in Bezug auf einen OP-Bericht *

*Markieren Sie nur ein Oval.*

- sehr gut
- gut
- befriedigend
- ausreichend
- mangelhaft

5. Inhalt (keine überflüssigen Details, enthält alle wichtigen Informationen, ist prägnant und umfasst das Wesentliche) *

*Markieren Sie nur ein Oval.*

- sehr gut
- gut
- befriedigend
- ausreichend
- mangelhaft

6. Lesefluss (lässt sich flüssig lesen, keine langen Schachtelsätze, leicht verständlich) *

*Markieren Sie nur ein Oval.*

- sehr gut
- gut
- befriedigend
- ausreichend
- mangelhaft

**Zeitaufwand**

7. Wie viel Zeit (min) wenden Sie ungefähr pro Tag für die Erstellung von OP- Berichten auf? *

*Markieren Sie nur ein Oval.*

- 0-30min /Tag
- 30-60min /Tag
- 60-120min /Tag
- >120min /Tag

8. *Reevaluation:* Zeitersparnis (Wie viel Zeit (min) würden Sie im Schnitt mit dieser Art von schlagwortbasierter, intraoperativer Berichterstellung pro Tag sparen?) *

*Markieren Sie nur ein Oval.*

- Keine Zeitersparnis (0 Minuten pro Tag)
- 1-15 Minuten pro Tag
- 16-30 Minuten pro Tag
- 31-45 Minuten pro Tag
- 46-60 Minuten pro Tag
- 61-90 Minuten pro Tag
- 91-120 Minuten pro Tag
- mehr als 120 Minuten pro Tag

**Mehrwert und Nutzen**

Bitte beziehen Sie sich auf den Gesamteindruck der OP-Berichte nach Ihrer Korrektur.

9. Arbeitslastreduktion (Workload-Entlastung, Stressreduktion, Zeitersparnis – Sehen Sie in dem Tool eine Reduktion der Arbeitslast?) *

*Markieren Sie nur ein Oval.*

- Ich stimme voll und ganz zu
- Ich stimme eher zu
- Ich stimme teilweise zu
- Ich stimme eher nicht zu
- Ich stimme überhaupt nicht zu

10. Mehrwert und Nutzen (Sehen Sie einen Nutzen in dieser neuen Art der Berichterstellung?) *

*Markieren Sie nur ein Oval.*

- Ich stimme voll und ganz zu
- Ich stimme eher zu
- Ich stimme teilweise zu
- Ich stimme eher nicht zu
- Ich stimme überhaupt nicht zu

11. Würden Sie in Zukunft dieses Tool für die OP-Berichterstellung nutzen? *

*Markieren Sie nur ein Oval.*

- Ich stimme voll und ganz zu
- Ich stimme eher zu
- Ich stimme teilweise zu
- Ich stimme eher nicht zu
- Ich stimme überhaupt nicht zu

**Vergleich zu den konventionellen OP-Berichten**

Bitte beziehen Sie sich auf den Gesamteindruck aller drei OP-Berichte nach Ihrer Korrektur.

12. Inhalt (Die Originalberichte und die erzeugten Berichte ähneln sich stark in der inhaltlichen Qualität) *

*Markieren Sie nur ein Oval.*

- Ich stimme voll und ganz zu
- Ich stimme eher zu
- Ich stimme teilweise zu
- Ich stimme eher nicht zu
- Ich stimme überhaupt nicht zu

13. Form/ Grammatik (Die Originalberichte und die erzeugten Berichte ähneln sich stark in der formalen Qualität) *

*Markieren Sie nur ein Oval.*

- Ich stimme voll und ganz zu
- Ich stimme eher zu
- Ich stimme teilweise zu
- Ich stimme eher nicht zu
- Ich stimme überhaupt nicht zu


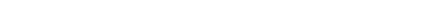

Supplement: Supplementary file 2 — Supplementary file2 (DOCX 24 kb) [file 11548_2022_2791_MOESM2_ESM.docx]
